# Exploration of Hypertension Following Traumatic Renal Hematoma Formation and Page Kidney Discussion

**DOI:** 10.7759/cureus.60468

**Published:** 2024-05-16

**Authors:** Caterina A Lind, Lindsay Tjiattas-Saleski

**Affiliations:** 1 Emergency Medicine, Edward Via College of Osteopathic Medicine, Spartanburg, USA

**Keywords:** secondary hypertension, traumatic hematoma, tpa liquification, ct-guided iv drainage, page kidney

## Abstract

Page kidney is defined as a rare cause of secondary hypertension due to a subcapsular hematoma externally compressing the kidney resulting in the activation of the renin-angiotensin-aldosterone system (RAAS). This phenomenon consists of numerous etiologies including acute or chronic traumatic or non-traumatic events. In this case, we report on an acute unilateral hematoma following blunt renal trauma as the result of a fall from standing height treated with tissue plasminogen activator (tPA) infusion and image-guided drainage. Qualities within this case and how they are paralleled in the diagnosis of a Page kidney are explored. A brief review of current etiologies and management plans per the literature review will also be discussed.

## Introduction

Page kidney is defined as a rare cause of secondary hypertension due to the external compression of the kidney by a subcapsular hematoma, of either traumatic or non-traumatic origins, activating the renin-angiotensin-aldosterone system (RAAS) [1]. Activation results in hypertension occasionally resistant to commonly used single or dual drug therapies of either angiotensin-converting-enzyme (ACE) inhibitors with or without beta-blockers [2]. Since its recognition in 1955, only a little over 100 cases of Page kidney have been reported. This is of note as blunt trauma is one of the most common reasons for emergency room visits today [3]. The abdomen, specifically, is currently the third most injured region of the body with approximately 25% of cases requiring surgery [3]. A Page kidney as the result of blunt trauma commonly requires a surgical approach consisting of image-guided drainage to relieve the kidney of the subcapsular hematoma’s mass effect [2]. The approach stated has shown to be effective over recent years, allowing for patients today to walk away with minimal, if any, long-term complications. This is in stark contrast to the previously accepted treatment plan consisting of a complete nephrectomy resulting in a rigorous follow-up and possible treatment regimen [2]. This case reports on an acute unilateral hematoma following blunt renal trauma treated with tissue plasminogen activator (tPA) infusion and image-guided drainage. Qualities within this case and how they are paralleled in the diagnosis of a Page kidney are explored. Lastly, current etiologies, pathophysiology, and management plans of Page kidneys and similar cases per the literature review will also be discussed.

## Case presentation

A 54-year-old male with a past medical history of sleep apnea-induced hypertension controlled with amlodipine 5 mg and hydrochlorothiazide-losartan 25-100 mg daily presented with complaints of left flank pain following a fall in his backyard the day before. The patient reported that the incident occurred accidentally while he was doing yard work and resulted in a direct impact to his left flank, minimally blunted by the ipsilateral arm and shoulder. The pain became worse throughout the night, leading to patient presentation. Upon arrival in the emergency department, the patient developed nausea and lightheadedness, followed by a brief syncopal episode witnessed and treated by medical personnel. No head trauma was observed, and a bedside-focused assessment with sonography was completed by an attending physician with negative results. On initial presentation, the patient’s blood pressure and heart rate were 143/71 mmHg and 84 bpm, respectively. Labs were completed, showing pertinent findings as follows (Table 1).

**Table 1:** Pertinent lab values upon evaluation and admission.

Test	Patient’s values	Reference values
Hemoglobin (Hgb)	11.7 g/dL	13.2–16.6 g/dL
BUN	30 mg/dL	5–20 mg/dL
Creatinine	1.7 mg/dL	0.6–1.2 mg/dL
Sodium (Na)	124 mmol/L	135–145 mmol/L

**Figure 1:**
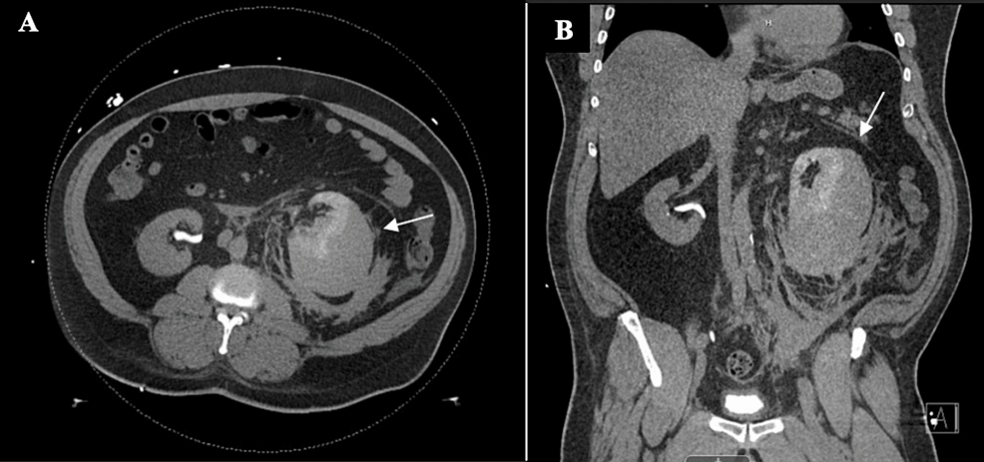
CT of the abdomen and pelvis without contrast. An enlarged left kidney and perinephric inflammation with subcapsular hematoma formation in the axial (a) and coronal (b) planes.

A computed tomography (CT) of the abdomen and pelvis without contrast was performed to identify the cause of flank pain (Figure 1). The predominant finding on this examination, as shown in Figure 1, was delayed left-sided nephrogram with large subcapsular fluid collection compressing the left kidney. Previous perinephric inflammation was also identified, extending along the left retroperitoneum. The left ureter is identified and does not appear to be obstructed.

**Figure 2:**
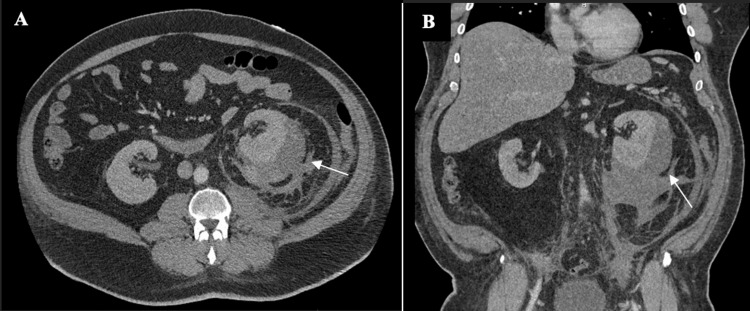
CT of the abdomen and pelvis with contrast. Subcapsular hematoma around the left kidney appears slightly increased in size (a). Pigtail drainage catheter is present within the subcapsular hematoma (b).

Ceftriaxone and intravenous (IV) fluids were administered empirically. Interventional radiology was emergently consulted and placed a CT-guided percutaneous left subcapsular drain one day after the presentation. On the day of drain placement, a blood pressure of 158/99 mmHg was recorded. A minimal amount of fluid from the hematoma was drained, indicating organization of the compressive mass, so tPA infusion was indicated for fibrinolysis. Upon successful liquefication, a total of 1.25L of fluid over the course of three days was drained. Blood pressure readings began to trend downward into the systolic 140s over 80s mmHg. Hemoglobin subsequently dropped to 8.1 g/dL and the creatinine increased to 1.8 mg/dL. Transfusion was not indicated at that time. Drainage of the hematoma continued for a total of nine days, with the utilization of tPA to aid in fibrinolysis for eight of those days. During this time, an increase in blood pressure of 165/95 mmHg was noted on day seven of admission. Blood pressure readings remained at systolic 150s over 90s mmHg for the two days following. To explore this finding, a CT of the abdomen and pelvis with contrast was ordered (Figure 2). A moderate subcapsular hematoma around the left kidney persisted and appeared slightly increased in size when compared to prior examination. Overall, the associated mass effect upon the left kidney appeared unchanged with near normal, symmetric enhancement compared to the right, as shown in Figure 2. The pigtail drainage catheter was present within the subcapsular hematoma on imaging as well.

As it was noted that the hematoma was still draining on the current treatment regimen, treatment was continued for three days. Upon continued drainage, the hemoglobin dropped to 6.6 g/dL one day prior to discharge, requiring a transfusion of one unit of packed red blood cells. Blood pressure readings also trended down following continued drainage, with a final reading of 143/78 mmHg on discharge. No antihypertensive medications were ordered during the duration of hospital admission, including previously prescribed medications, and the pain was managed with acetaminophen, hydromorphone, and tramadol as needed. The patient was ultimately discharged with his drain in place after a 10-day stay with instruction to continue his chronic medication regimen of amlodipine 5 mg and hydrochlorothiazide-losartan 25-100 mg daily and start tadalafil 20 mg prn, tramadol 50 q6h prn, brompheniramine/dextromethorphan/PSE prn, and topical docosanol. The patient presented as an outpatient approximately four days post-discharge for drain removal. Two weeks following removal, a renal ultrasound and basic metabolic panel were completed and returned normal. To date, the patient reports no chronic sequelae and maintains sleep apnea-induced hypertension treated with previously stated home medications and continuous positive airway pressure (CPAP) use.

## Discussion

Etiology

First described in 1939, the “Page” kidney was explored through wrapping of an animal’s kidney in cellophane resulting in the formation of dense, compressive scar tissue [4]. Experimental reproduction of secondary hypertension led to the deduction that organizing hematomas of varying etiologies could do the same [4]. Medical etiologies primarily consist of bleeds from blunt trauma, renal procedures, and surgeries (e.g., biopsy, transplants, and lithotripsy), or of spontaneous origin (e.g., arteriovenous malformations, tumor, and anticoagulation) [5]. Less common nonbleeding causes can consist of lymphoceles, cysts, and urinomas [6]. The kidney consists of a solid organ enwrapped in a fibrous capsule layer separating an overlaying fascia [1]. Because the capsule layer lines the kidney closely, even a small hemorrhage under the renal capsule can cause hemostatic instability [7]. By contrast, as there is a large space between the renal fascia and capsule, a sizable hematoma is necessary to compress the kidney [5]. The latter description of extracapsular hemorrhagic compression is the cause of most Page kidney cases reported [5].

Pathophysiology

The RAAS mediates the characteristic secondary hypertension in the Page kidney [5]. Activation develops when extrinsic compression of the renal arteries by the hematoma causes a decrease in afferent blood flow [1]. This decrease is sensed by the organ, initiating the release of renin, the enzyme necessary to convert angiotensinogen to angiotensin-I [8]. Once produced, the angiotensin-converting enzyme then continues the cascade by catalyzing the conversion of angiotensin-I to angiotensin-II [8]. Numerous physiological effects occur from this conversion, including vasoconstriction of arterioles and increased water retention via vasopressin release from the hypothalamus [8]. These effects, along with the increase in aldosterone production by the adrenal cortex resulting in additional renal sodium reabsorption, lead to increased systemic vascular resistance and thus the notorious secondary hypertension of a Page kidney [1].

Discussion

Page kidneys and related cases as the result of blunt trauma can be assessed and treated in a variety of ways. In this case, while the initial imaging modality withheld contrast, the subsequent imaging involved an IV contrast-enhanced CT imaging. This modality is recommended by the American Association for the Surgery of Trauma to assess renal trauma [9]. Magnetic resonance imaging (MRI) may also help in assessing the organization and age of the hematoma along with the patency of the vessels it is compressing [10]. A Doppler ultrasound can also be utilized to aid in identifying the impact of the hematoma on renal blood flow per the renal arterial resistive index value [11]. Once identified, the age and organization of the subcapsular hematoma will dictate the most appropriate treatment plan. In objectively mild cases, medical management consisting of angiotensin-converting enzyme inhibitors is trialed first [10]. Simultaneously, inpatient observation of hematoma absorption is done to determine resolution [9].

Surgical management consisting of percutaneous image-guided drainage, surgical drainage, and capsulotomy can be trialed for more severe cases [1]. As these options are minimally invasive and result in decreased rates of morbidity, they are increasingly becoming first-line treatment plans, as exemplified in this case [6]. In patients presenting with organized or treatment-resistant hematomas, definitive surgery options consist of capsulectomy, partial nephrectomy, or total nephrectomy [10]. According to the American Urological Association, a large perirenal hematoma (>4 cm) in a hemodynamically unstable patient would require immediate surgical intervention [12]. As Page kidneys and similarly acting hematomas are such a rarity, the treatment plan that is most beneficial in preventing morbidity and mortality is still being debated. A retrospective case series analyzing a total of 26 Page kidneys of traumatic and non-traumatic origin determined inconsistencies in medication use due to changing medication guidelines, while nephrectomies prevailed as the treatment of choice in nontraumatic cases [13].

In this case, the substantial organization of the subcapsular hematoma was determined to best be treated with percutaneous image-guided drainage via tPA infusion. No antihypertensive medical management was utilized alongside the stated treatment plan or needed chronically other than those already prescribed previously. Other reported cases have shown that organized hematomas are typically not amended by percutaneous drainage due to their consistency [5]. With the utilization of tPA aiding in liquefication, this treatment modality showed itself to be successful and minimally invasive.

## Conclusions

Page kidney is a rare phenomenon resulting in secondary hypertension and can be treated in numerous ways. In the case of organized hematomas, medical management is commonly insufficient, and invasive measures are required. Minimally invasive image-guided percutaneous drainage, with the use of tPA for liquefaction purposes, can be performed to successfully reduce organized hematomas. This is important to acknowledge as treatment of Page kidney and related cases is still provider-dependent. With this case, we hope to raise awareness of tPA liquefaction use and the negligible treatment-associated morbidity it provides for future guidance.
